# Tandem Expression of a Mobile RNA and Its RNA-Binding Protein(s) Enhances Tuber Productivity in Potato

**DOI:** 10.3390/ijms242115754

**Published:** 2023-10-30

**Authors:** Kirtikumar R. Kondhare, Nikita S. Patil, Sundaresha Siddappa, Anjan K. Banerjee, David J. Hannapel

**Affiliations:** 1 Biology Division, Indian Institute of Science Education and Research (IISER), Homi Bhabha Road, Pune 411008, Maharashtra, India; kr.kondhare@ncl.res.in (K.R.K.); patil.nikita@students.iiserpune.ac.in (N.S.P.); 2 Crop Improvement Division, Central Potato Research Institute (CPRI), Shimla 171001, Himachal Pradesh, India; 3 Plant Biology Major, 253 Horticulture Hall, Iowa State University (ISU), Ames, IA 50011, USA

**Keywords:** BEL5, tuber productivity, long-distance signaling, mobile signal, PTB

## Abstract

A significant number of discoveries in past two decades have established the importance of long-distance signaling in controlling plant growth, development, and biotic and abiotic stress responses. Numerous mobile signals, such as mRNAs, proteins, including RNA-binding proteins, small RNAs, sugars, and phytohormones, are shown to regulate various agronomic traits such as flowering, fruit, seed development, and tuberization. Potato is a classic model tuber crop, and several mobile signals are known to govern tuber development. However, it is unknown if these mobile signals have any synergistic effects on potato crop improvement. Here, we employed a simple innovative strategy to test the cumulative effects of a key mobile RNA, *StBEL5*, and its RNA-binding proteins, StPTB1, and -6 on tuber productivity of two potato cultivars, *Solanum tuberosum* cv. Désirée and subspecies *andigena*, using a multi-gene stacking approach. In this approach, the coding sequences of *StBEL5* and *StPTB1/6* are driven by their respective native promoters to efficiently achieve targeted expression in phloem for monitoring tuber productivity. We demonstrate that this strategy resulted in earliness for tuberization and enhanced tuber productivity by 2–4 folds under growth chamber, greenhouse, and field conditions. This multi-gene stacking approach could be adopted to other crops, whose agronomic traits are governed by mobile macromolecules, expanding the possibilities to develop crops with improved traits and enhanced yields.

## 1. Introduction

Long-distance macromolecular signaling enables plants to modulate developmental events in response to environmental cues at the whole-plant level. Numerous mobile signals (mRNAs, small RNAs, proteins, small peptides, phytohormones, etc.) ferry the phloem cell system and govern a range of physiological activities [1,2,3,4,5,6,7,8,9,10]. Mobile signals have been identified in *Arabidopsis thaliana*, potato (*Solanum tuberosum*), tomato (*Solanum lycopersicum*), pumpkin (*Cucurbita maxima*), melon (*Cucumis melo*), wheat (*Triticum aestivum*), rice (*Oryza sativa*), maize (*Zea mays*), pear (*Pyrus communis*), and apple (*Malus domestica*) that regulate sink formation and impact crop productivity. For example, phloem-mobile microRNAs regulate grain size, shape, and quality in cereals [11,12]. In developing rice endosperm, OsRBP-P (RNA-BINDING PROTEIN-P) facilitates the transport of *GLUTELIN* and *PROLAMINE* mRNAs through the endoplasmic reticulum to regulate kernel development [13]. In *Arabidopsis*, a mobile transcription factor (TF), HY5, coordinates carbon assimilation and improves the nutrient-use efficiency of plants [14,15]. mRNAs from the *FLOWERING LOCUS T (FT)* clade function as long-distance signals to regulate flowering [16,17,18]. The RNA-BINDING PROTEIN (RBP), CmRBP50, was identified from the pumpkin phloem sap and forms a ribonucleoprotein (RNP) complex containing six mRNAs and sixteen proteins [19]. This complex provides stability and facilitates RNA transport. Although numerous mRNAs are detected in the phloem sap [20,21,22,23,24], only a few select mRNAs are known to have physiological relevance [25,26,27,28,29,30,31].

**Figure 1:**
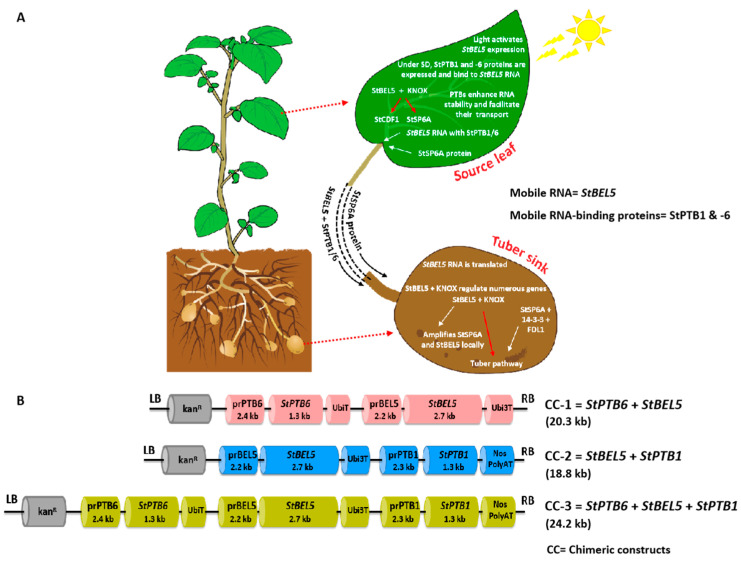
Schematics of potato tuberization pathway driven by mobile factors and design of chimeric constructs. (**A**) Illustration of potato tuberization pathway driven by a mobile RNA (*StBEL5*), StSP6A protein, and RNA-BINDING PROTEINS (StPTB1/6) in response to photoperiod. (**B**) Schematics of the chimeric constructs (CCs): CC-1, CC-2, and CC-3. The plasmid sizes of CCs containing the binary vector backbone were 20.3 kb, 18.8 kb, and 24.2 kb for CC-1, CC-2, and CC-3, respectively. Abbreviations: pr, Promoter; KanR, Kanamycin resistance cassette; kb, Kilobase; LB, Left border; *NosT, NOPALINE SYNTHASE TERMINATOR; NPTII, NEOMYCIN PHOSPHOTRANSFERASE*; RB, Right border; *Ubi, UBIQUITIN; UbiT, UBIQUITIN TERMINATOR; Ubi3T; UBIQUITIN 3 TERMINATOR*.

Several studies in the past two decades have established potato as a valuable model system for studying the phloem-mobile signals regulating storage organ development [32,33,34,35]. The first phloem-mobile mRNA in potato, *StBEL5*, a BEL-like TF moves from leaf to stolon (a belowground modified stem) to regulate tuberization [32] (Figure 1A). In stolons, StBEL5 works in tandem with a KNOTTED1-like (KNOX) TF to control the expression of key tuberization genes [36]. This tandem complex regulates the expression of the FT ortholog *StSP6A* [34] to mediate the onset of tuber formation [37,38,39] (Figure 1A). Movement and stability of *StBEL5* is shown to be controlled by two RBPs from the family of POLYPYRIMIDINE TRACT-BINDING (PTB) proteins, designated as *StPTB1* and -6. These PTB proteins bind to the *StBEL5* mRNA to form an RNP complex present in the phloem [40] (Figure 1A). The mobility of *StBEL5* RNA and a significant function of specific StPTBs is crucial in protecting, transporting, and localizing its transcript and controlling its function. Earlier, we have demonstrated that the individual overexpression of *StBEL5* or *StPTB1* or *StPTB6* results in enhanced tuber productivity in potato [32,40]. However, if tandem expression of these mobile signals has any cumulative effects on tuber productivity have not yet been tested. In this study, we employed a multi-gene approach using a key mobile RNA, *StBEL5*, and its RNA-binding proteins, *StPTB1* and -6, driven by their respective native promoters and demonstrate up to 4-fold tuber weight increase under both greenhouse and field conditions. The strategy is simple and effective in the improvement of tuber productivity in potato and could have wider applications in other crops.

## 2. Results

### 2.1. Assessment of Earliness for Tuberization under In Vitro and Greenhouse Conditions

**Figure 2:**
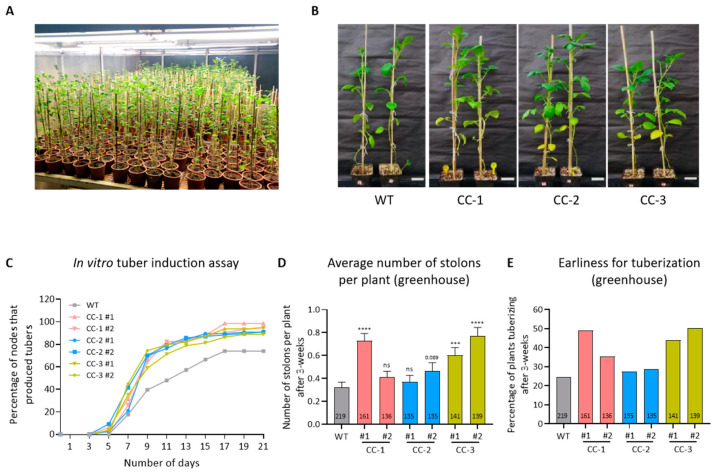
Evaluation of earliness for tuberization in Désirée CC lines. (**A**) Image of wild-type (WT) and CC lines of *S. tuberosum* cv. Désirée growing in the greenhouse. (**B**) Representative images of WT and CC Désirée lines after 12 weeks of transfer to soil. Lower yellow leaves in CC lines indicate their earliness for maturity. The enhanced expression of *StCDF1* in these lines (shown later in Figure 3A) could be the reason. Scale bar = 5 cm. (**C**) In vitro tuber induction experiment (8% sucrose) showed an earliness in transgenic CC Désirée lines compared to WT. (**D**) Average number of stolons per plant after 3-week of transfer to greenhouse. Student’s *t*-test was used at *p* < 0.05 (*** *p* < 0.001, **** *p* < 0.0001, ns, not significant). Mean values ±SEM are represented in the graph. (**E**) Evaluation of earliness for tuberization in greenhouse after 3-weeks. The graph represents the percentage of plants that formed stolons and/or swollen stolon after three weeks of growth. For panels (**C**,**E**), no statistical test is performed as the data is plotted as the percentage of nodes or plants tuberizing. For panels (**D**,**E**), values inside the bars represent the number of plants per line (*n*). #1 and #2 are the two independent lines of respective CC used throughout the studies (as mentioned in Supplementary Figure S1).

To validate our hypothesis, chimeric constructs (CCs) were designed that express *StBEL5* mRNA and the two StPTB proteins in tandem and are driven by their respective native promoters (Figure 1B). These constructs (CC-1, CC-2, and CC-3) utilizing a combination of *StBEL5* plus its RNA-BINDING PROTEINS, *StPTB1* and -6, were cloned into a pCAMBIA binary vector, followed by mobilization to *Agrobacterium tumefaciens* strain GV2260 (Figure 1B). These chimeric constructs were used for the generation of several transgenic CC lines in Désirée and two select lines per chimeric construct were used for further experiments (Figure 2A,B; Supplementary Figure S1). We selected potato cultivar Désirée because it is a day-neutral plant and can be grown in the greenhouse under normal long-day as well as in the field conditions of India. These multi-gene lines, designated as CC-1, CC-2, and CC-3, exhibited growth and shoot architecture comparable to WT having no phenotypic abnormalities (Figure 2B).

To assess the rate of tuber formation in the Désirée CC lines, an in vitro tuber induction assay (supplemented with 8% sucrose) was performed. These lines exhibited 2-day earliness (day 3 versus day 5) for tuber formation compared to WT, except CC-1 #2 and CC-3 #2 lines, which recovered in the subsequent four days (Figure 2C). On day 9, up to 40% nodes tuberized from WT, whereas the number of tuberizing nodes varied between 59 to 75% for CC lines. After 21 days, 89–99% nodes of CC lines had formed tubers as opposed to 74% nodes of WT (Figure 2C). The CC lines were also grown in soil to assess their rate of tuber formation under greenhouse conditions. After three weeks of transfer to soil, they had more stolons per plant compared to WT (Figure 2D) and exhibited earliness for tuberization (Figure 2E), consistent with the observations of the in vitro tuberization experiment.

### 2.2. Expression Analysis of Tuber Marker Genes and Evaluation of Tuber Productivity under Controlled Growth Conditions

**Figure 3:**
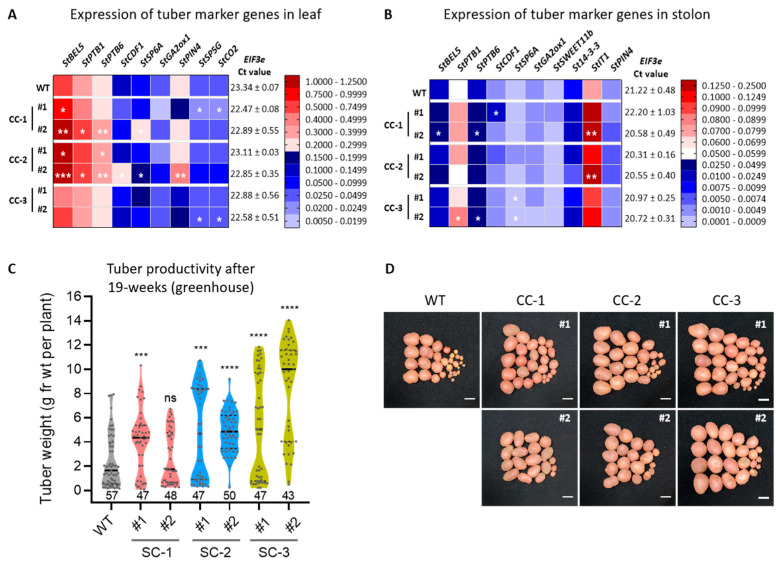
Expression of tuber marker genes and assessment of tuber productivity in Désirée CC lines under greenhouse conditions. Heat maps depict the relative expression of key tuberization genes in leaves (**A**) and stolons (**B**) of 3-week-old greenhouse plants. Data represents mean of three biological and three technical replicates. Potato *EIF3e* was used for normalization of gene expression and Ct values are represented next to the respective rows. #1 and #2 are the two independent lines of respective CC used throughout the studies (as mentioned in Supplementary Figure S1). (**C**) Average tuber weight in transgenic Désirée CC lines from 19-week-old plants from greenhouse. The numbers below violin plots represent the number of plants per line (n). In the respective violin plot, a thick central line represents a median and the dotted lines indicate the lower and higher quartiles of the corresponding data points. In panels (**A**–**C**), Student’s *t*-test was used at *p* < 0.05 (* *p* < 0.05, ** *p* < 0.001, *** *p*< 0.005, **** *p* < 0.0001, ns, not significant). (**D**) Tuber images from greenhouse plants. For imaging, tubers were pooled from 15 plants for each line. Scale bar = 2 cm. The experiment for tuber productivity from greenhouse grown plants was performed two times and similar observations were found. The current data is from the second experiment. Abbreviations: *BEL*, *BEL1-LIKE* transcription factor; *CDF1, CYCLING DOF FACTOR*; *CO2, CONSTANS2*; *GA2OX1, GIBBERELLIN 2 OXIDASE 1*; *IT1, IDENTITY OF TUBER 1*; *PIN4, PIN FORMED 4*; *PTB1/6, POLYPYRIMIDINE TRACT-BINDING PROTEINS 1/6*; *SP5G, SELF-PRUNING 5G*; *SP6A, SELF-PRUNING 6A*; *SWEET11B, SUGARS WILL EVENTUALLY BE EXPORTED TRANSPORTER 11B*.

After three weeks of growth in greenhouse, leaves and stolons of Désirée CC lines and WT plants were harvested for expression analysis of tuber marker genes. Expression profiles of select tuber marker genes revealed an upregulation of *StSP6A* (tuber initiation marker) and *CYCLING DOF FACTOR 1* (*StCDF1*; an earliness marker) in leaves of transgenic CC lines compared to WT (Figure 3A). In stolons, *St14-3-3* and *PIN-FORMED 4* (*StPIN4*) were upregulated in CC lines (Figure 3B). RNA levels of *StGA2ox1* exhibited a modest increase. *GIBBERELLIN 2-OXIDASE 1* (*StGA2ox1*) is an important tuber gene. Its expression is activated by StBEL5 [36] and it promotes tuberization by degrading gibberellins just prior to stolon swelling [41]. The *TUBER IDENTITY* gene *StIT1* was also found to be significantly upregulated in the stolons of CC lines. *StIT1* is key to the initiation of tuberization and interacts with *StSP6A* to promote tuber development [42]. These findings support the premise of early tuber induction in CC lines.

The tuber productivity of Désirée CC lines was assessed after they reached physiological maturity (19 weeks in greenhouse). The CC lines demonstrated a significant increase in tuber weight per plant ranging from 1.70 to 3.54-fold compared to WT, except CC-1 #2 (Figure 3C). Tubers produced from these lines had similar morphology as that of WT. Though the tuber numbers did not vary much compared to WT, the tuber size appeared to be larger for CC lines (Figure 3D).

**Figure 4:**
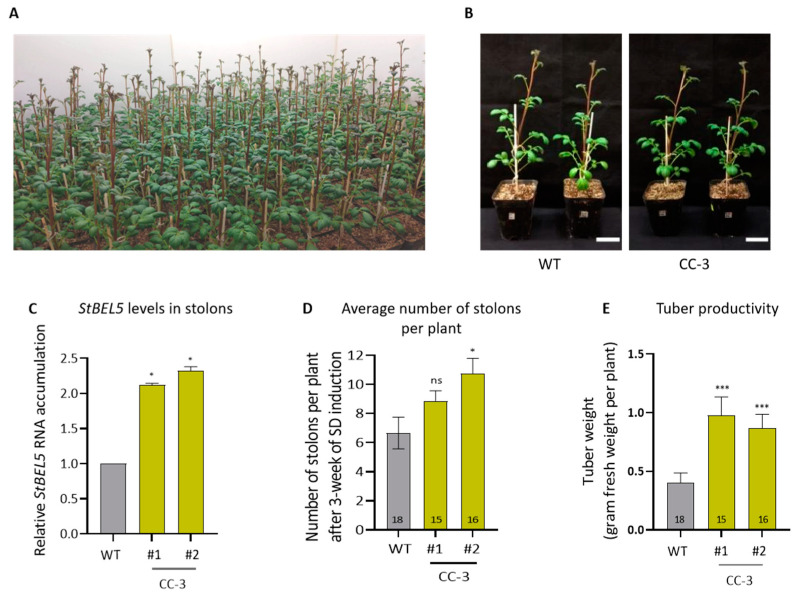
Assessment of tuber productivity in *andigena* CC lines under SD photoperiod. (**A**) Image of wild-type (WT) and CC-3 lines of *S. tuberosum* ssp. *andigena* (a photoperiod-responsive ssp.) growing in the plant growth chamber. (**B**) Representative images of WT and CC-3 *andigena* lines taken after 8 weeks of transfer to soil. Scale bar = 5 cm. (**C**) Relative levels of *StBEL5* RNA in stolons of transgenic CC-3 *andigena* lines compared to WT. Potato *EIF3e* was used for normalization of gene expression. For RT-qPCR analysis, three biological replicates and three technical replicates were used (*n* = 3). (**D**) Average number of stolons per plant after 3 weeks of SD induction in plant growth chamber. Student’s *t*-test was used at *p* < 0.05. ns = not significant. (**E**) Tuber productivity of transgenic CC-3 *andigena* lines in comparison to WT after 3 weeks of SD photoperiod. For panels (**D**,**E**), values inside the bars represent the number of plants per line (n). For panels (**C**–**E**), values are represented as mean ±SEM. Student’s *t*-test was used at *p* < 0.05 (* *p* < 0.05, *** *p* < 0.001, ns, not significant). #1 and #2 are the two independent CC-3 *andigena* lines used in this study (as mentioned in Supplementary Figure S2).

To test the effectiveness of our strategy in another potato cultivar, we generated seven transgenic CC-3 lines of *S. tuberosum* ssp. *andigena* (Figure 4A,B; Supplementary Figure S2). *Andigena* was selected because this cultivar is responsive to photoperiod [32]. Similar to Désirée CC lines, *andigena* CC-3 lines (#1 and #2) had elevated levels *StBEL5* in stolons (Figure 4C). These *andigena* lines produced more stolons per plant (Figure 4D) and had enhanced tuber productivity under SD conditions in comparison to WT (Figure 4E).

### 2.3. Evaluation of Tuber Productivity under Field Conditions

**Figure 5:**
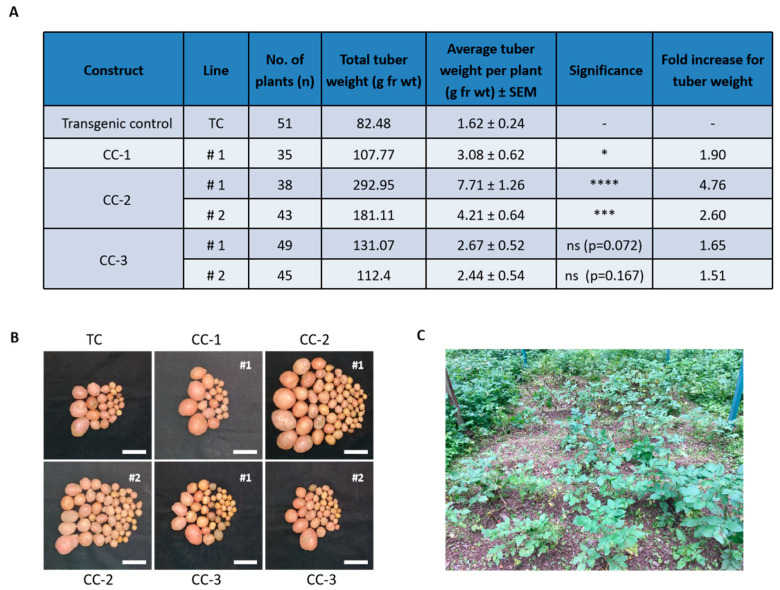
Evaluation of tuber productivity in Désirée CC lines under field conditions. (**A**) Tuber harvest data of the transgenic Désirée CC plants grown in field conditions for 19 weeks at the ICAR Central Potato Research Institute (CPRI) located at Shimla, India. (**B**) Tuber images of transgenic control and Désirée CC lines. Scale bar = 5 cm. (**C**) Image of transgenic plants growing in the field conditions at CPRI. The field experiment was conducted two times in the year of 2021 and 2022 and similar results were obtained. #1 and #2 are the two independent lines of respective CC used throughout the studies (as mentioned in Supplementary Figure S1). For panel (**A**), Student’s *t*-test was used at *p* < 0.05 (* *p* < 0.05, *** *p* < 0.001, **** *p* < 0.0001, ns, not significant). SEM = Standard error of mean. TC = Transgenic control. Figure contains the data obtained from the second field experiment.

To evaluate the performance of Désirée CC lines under field conditions, we grew them in a contained facility at ICAR Central Potato Research Institute (CPRI), Shimla (Figure 5A–C). These CC lines showed an average 2-fold increase in tuber weight per plant compared to transgenic control (Figure 5A). Depending on the construct used, CC lines exhibited increased tuber productivity in the range of 1.50 to 4.76-fold. Among the three CC lines, CC-2 plants (line #1 and #2) showed highest tuber productivity. The morphology of tubers from all CC lines were comparable to transgenic control (Figure 5B) and produced viable sprouts following dormancy.

## 3. Discussion

Tuberization in potato is a complex process governed by various intrinsic and extrinsic factors. Numerous signals, such as *StBEL5*, *StBEL11*, *StBEL29*, *POTH1*, *StPTB1*, *StPTB6*, microRNA156, microRNA172, and *StSP6A*, are pivotal in the governance of tuber development [39]. Individual overexpression of *StBEL11*, *StBEL29*, or miR156 is shown to have a negative effect on tuber formation [39], whereas that of *StBEL5*, *StPTB1*, *StPTB6*, microRNA172, and *StSP6A* positively regulate tuberization [32,33,34,40]. *StBEL5* mRNA is the first mobile signal discovered in potato and it belongs to the Three-Amino acid-Loop-Extension (TALE) superfamily of TFs [32]. Under tuber-inductive conditions, the potato RBPs (*StPTB1* and -6) bind to cytosine/uracil-rich sequences present in the 3′ UTR of the *StBEL5* RNA to form a RNP complex that facilitates the stability and targeted delivery of *StBEL5* transcripts from leaf to stolon, the site of tuber formation [40]. Along with its KNOX partner, *StBEL5* regulates key tuberization genes: *StSP6A* (tuber initiation marker), *StCDF1* (plant maturity), *GIBBERELLIN 20-OXIDASE 1* (*StGA20ox1*), *GIBBERELLIN 3-OXIDASE 2* (*StGA3ox2*), *GIBBERELLIN 3-OXIDASE 3* (*StGA3ox3*), *GIBBERELLIN 2-OXIDASE 1* (*StGA2ox1*; gibberellin metabolism), *ISOPENTENYL PYROPHOSPHATE TRANSFERASE* (*StIPT*; cytokinin biosynthesis), *StYUCCA4* (auxin biosynthesis), and several other genes [36,43]. In addition, *StBEL5* auto-regulates its own expression to amplify the signals in stolon [36] and, in this way, acts as an important regulator of tuber development in potato.

The constitutive expression of *StBEL5* mRNA has consistently produced a positive effect on tuber formation [32]. Individual overexpression of the two PTBs (*StPTB1* or -6) indirectly leads to enhanced tuber productivity by increasing *StBEL5* transcript levels in the stolon [40]. These early experiments demonstrate increased productivity in the transgenic Désirée and ssp. *andigena* lines utilizing the constitutive 35S CaMV promoter or a leaf-specific promoter [32,40,44,45]. In addition, in a few instances, mislocalization of *StBEL5* transcripts either due to its ectopic expression (under 35S CaMV promoter) or the lack of untranslated regions (UTRs) in the coding sequence (CDS) caused an induction of aerial stolon/tubers accompanied with reduced tuber weight [32,44,45]. Therefore, to achieve optimum expression in this dual system, it appears crucial to retain the native spatio-temporal accumulation patterns and localization of *StBEL5* transcript to the target organs.

To test the cumulative effects of the tandem expression of *StBEL5* and/or *StPTB1/6* on tuber productivity in potato, we designed three CCs. To closely mimic the wild-type expression profiling of these genes, native promoters were included in these CCs to efficiently drive their targeted, localized expression in the vasculature of phloem cells, stolons, and leaf veins [32,40]. To achieve efficient transport of *StBEL5*, its full-length transcript (CDS + UTRs) was used in all three CCs. Our rationale in this combinatorial system was that an increase in both of these components in the phloem system would enhance the delivery of *StBEL5* to stolons, thereby creating a strong tuber sink that would lead to a positive effect on tuber productivity.

Stable transgenic CC lines of a day-neutral potato cv. Désirée exhibited earliness for tuberization under in vitro and greenhouse conditions accompanied with an increased number of stolons per plant, suggesting that effective delivery of *StBEL5* to stolons potentiates the plant for increased tuber productivity. As expected, an upregulation of several tuber marker genes (*StCDF1*, *StSP6A*, *StPIN4*, and *StIT1*) in leaves and stolons of these CC lines was observed. These expression profiles suggest that *StBEL5* and its PTB partners are working in consonance to activate components of the downstream tuber pathway. Notably, we found an increase of 2.1-fold tuber weight per plant in Désirée CC lines compared to WT. These results were comparable to previously observed transgenic lines containing *StBEL5* or *StPTB* genes driven by the 35S CaMV promoter or a leaf-specific GAS promoter [32,40,44,45], suggesting that our strategy of utilizing native promoters in a multi-gene approach is equally effective as that of previously employed constitutive expression approaches. Functionality of the CC-3 construct in *andigena* ssp. further validates that the multi-gene approach is consistent and robust and could readily be applied to other potato cultivars. The field evaluation of Désirée CC lines demonstrated that their average tuber weight per plant was enhanced by 2.5-fold when compared to the control. Though the fold increase in tuber weight of CC-3 lines (1.51 and 1.65) was not statistically significant, it still holds promise. The performance of CC lines under field conditions signifies the translational potential of our multi-gene approach for enhancing tuber productivity. Taken together, our results from the in vitro tuber induction, greenhouse, and field experiments clearly establish that the tandem expression of mobile signals (*StBEL5* and *StPTB1/6*) driven by their native promoters has exerted cumulative effects to increase tuber productivity in a major food crop, potato.

An exciting observation arising from our current study was the effective use of the multi-gene transformation (MGT) strategy to produce transgenic lines. The characteristics of our CCs are—(i) they contained multiple gene cassettes between the left and right border of a single T-DNA fragment (two or three effect genes: *StBEL5*, *StPTB1*, and/or *StPTB6* and one antibiotic marker), and (ii) the effect genes are plant-specific from potato, driven by their respective native promoters, and were not expressed ubiquitously or constitutively. MGT has been applied in mustard, soybean, canola, maize, *Arabidopsis*, potato, rice, and tobacco for regulating metabolic pathways and stress resistance [46,47,48,49,50,51,52,53,54,55,56,57,58]. Our current report documents a technical advance in demonstrating enhanced crop productivity utilizing mobile RNP components via a MGT approach.

Recent reports have identified the orthologs of potato mobile factors (*StBEL5*, *POTH1*, StPTB1/6, and StSP6A) in five storage root crops: sweet potato, cassava, carrot, radish, and sugar beet. In silico studies suggest that the RNA recognition motifs of RBPs, cytosine/uracil-rich sequences in the UTRs of target RNA orthologs, and the gene-regulatory network governed by mobile factors are also conserved between a tuber crop (potato) and these storage root crops [59]. Previous experiments have confirmed the role of phloem mobile *StBEL5* in enhancing root growth in potato [43]. Based on the functions of these orthologs and consistent with their potential role in root crop development, a similar tandem gene expression strategy could be utilized to increase the yield of storage root crops.

Apart from potato, RBPs governing reproductive traits are reported from *Arabidopsis* [60,61] and rice [13]. AtPTB1 and -2 regulate the mobility of *FLOWERING LOCUS K*, *FLOWERING LOCUS M*, *PHYTOCHROME-INTERACTING FACTOR 6* to control pollen and seed germination and flower development in *Arabidopsis* [60,61]. On the other hand, *GLUTELIN* and *PROLAMINE* are associated with OsRBP-P regulate kernel development in rice [13]. It remains to be seen whether the tandem expression of RNP components in rice could also increase its yield. This approach of synergistic expression of mobile macromolecular signals driven by their native promoters presents a novel strategy for engineering plants to enhance crop productivity and represents an excellent example of harvesting the translational knowledge gained from studying the fundamental processes of plant development.

## 4. Materials and Methods

### 4.1. Plantlet Source and Culture

In vitro plants of two potato cultivars (*Solanum tuberosum* cv. Désirée and ssp. *andigena* 7540) were multiplied from the axillary node sub-cultures on Murashige and Skoog’s basal medium [62] containing 2% (*w*/*v*) sucrose. In vitro plants were grown in a plant growth incubator (Percival Scientific, Inc., Perry, IA, USA) under long-day (LD; 16 h light and 8 h dark) conditions at 24 °C for three weeks and used for further experiments or maintained under the same conditions in vitro using the axillary node sub-cultures.

### 4.2. Chimeric Constructs Preparation

The full-length mRNA sequence of *StBEL5* (2716 bp; consisting of 147 bp 5′ untranslated region [UTR] and 503 bp 3′ UTR) was amplified from the petiole complementary DNA (cDNA) of *S. tuberosum* ssp. *andigena* 7540, whereas the *StBEL5* promoter sequence (2242 bp with last 55 bp of 5′ UTR deletion) was amplified from the shoot-tip genomic DNA. Similarly, the full-length coding sequence (CDS) of *StPTB1* (1338 bp) and *StPTB6* (1348 bp) were amplified from the petiole of *S. tuberosum* cv. Désirée plants, and their corresponding promoter sequences (*StPTB1*- 2301 bp and *StPTB6*- 2458 bp, including the respective introns from 5′ UTR) were amplified from genomic DNA isolated from the shoot-tip samples of soil-grown plants. All six sequences (three promoters and three CDSs) were individually cloned into the sub-cloning vector pGEM-T Easy (Promega Incorporation) and sequence verified. A combination of *StBEL5* and *StPTB1* and/or *StPTB6* CDS(s) were placed under their respective promoter sequences and three multi-gene chimeric constructs (CCs) were prepared in the pCAMBIA binary vector backbone. They are referred to as CC-1 (*StBEL5* + *StPTB6*; 20.3 kb), CC-2 (*StBEL5* + *StPTB1*; 18.8 kb), and CC-3 (*StBEL5* + *StPTB1* + *StPTB6*; 24.2 kb) (Figure 1B). These CC constructs were sequence verified and transformed into *Agrobacterium tumefaciens* strain GV2260.

### 4.3. Generation of Stable Transgenic Lines

Several transgenic potato lines of a day-neutral cultivar, Désirée (CC-1, CC-2, and CC-3) and a photoperiod-responsive ssp. *andigena* (only CC-3) were generated for CCs along with a transgenic control line as per the method described in [24]. The transgenic lines were confirmed by reverse transcription polymerase chain reaction (RT-PCR) using gene-specific forward primer and terminator-specific reverse primer (Table S1) as well as by reverse transcription quantitative PCR (RT-qPCR) analysis.

### 4.4. In Vitro Tuber Induction Assay

To evaluate the earliness for tuber formation in transgenic CC and wild-type (WT) Désirée plants, an in vitro tuberization experiment was conducted as per the method described in [63] with minor modifications. Single nodal explants (top four nodes per plant, excluding the shoot apex) from four weeks in vitro plantlets (grown under LD conditions) were sub-cultured on MS medium containing 8% sucrose (*w*/*v*; tuber-induction medium). Culture plates were incubated under dark conditions in a growth incubator maintained at 24 °C. The rate of tuber formation was scored on alternate days until 21 days. A minimum of 84 single node explants per line or WT were used for the experiment.

### 4.5. Tuber Productivity and Quantification of StBEL5 Transcript Levels

One month old plantlets of the selective transgenic CC lines (Désirée and ssp. *andigena*) along with respective WT were grown in vitro under LD conditions. The plantlets were transferred to soil in small size round pots (dimensions: height = 6.3 cm and radius = 3.5 cm; volume: ~242 cm^3^), and hardened for 1 week under LD conditions in a growth chamber (Percival Scientific, Inc.) with light intensity: 300 μmol m^−2^ s^−1^), day temperature 24 °C and night temperature 22 °C. This was followed by additional two weeks of incubation in the same pots. Thereafter, both types of cultivar lines were re-potted into the medium size pots (dimensions: height = 10.5 cm, and radius = 5.75 cm; volume: ~1090 cm^3^) and continued to grow under LD conditions for an additional 5 weeks. The tuber productivity (gram fresh weight [g fr wt] per plant) for Désirée lines was recorded after 19 weeks of plant growth (physiological maturity). When *andigena* plants attained the 10–13 leaf stage, they were transferred to short-day (SD) conditions (16 h dark and 8 h light) with 22 °C and 20 °C day and night temperatures, respectively. After 21 days of SD induction, these plants were scored for number of stolons and tuber weight. In an additional experiment, Désirée CC lines and WT plants were scored for the number of stolons and the percentage of plants tuberizing post three weeks of transfer to soil.

Stolons from all CC lines and WT *andigena* plants were harvested at the end of the experiment and used for quantification of *StBEL5* transcript levels. For Désirée plants, leaves and stolons from CC lines and WT (4 plants per replicate) were harvested for quantification of *StBEL5* transcript levels as well as key tuberization genes. RT-qPCR data represents the mean ± SEM (standard error of mean) of three biological and three technical replicates. Total RNA was isolated using RNAiso Plus (DSS-TAKARA) and the quality of RNA was checked on agarose gel electrophoresis. cDNA was synthesized using 2 µg of the total RNA, Superscript IV Reverse Transcriptase (Invitrogen) and oligo (dT) primers. RT-qPCR reactions were carried out on the CFX96 Real-Time System (BIO-RAD) with gene-specific primers and TAKARA SYBR^®^ green master mix (Takara-Clontech) by incubating at 95 °C for 3 min, followed by 40 cycles at 95 °C for 5 s, gene-specific annealing temperature for 15 s and 72 °C for 20 s. Data were analyzed using either the 2^–ΔΔCt^ relative fold-change or 2^−ΔΔCt^ relative abundance method [64]. Potato *EIF3e* was used as a reference for normalization of gene expression. Students *t*-test was performed to analyze the data from various experiments. One, two, three and four asterisks (*) represent the level of significance at *p* < 0.05, *p* < 0.01, *p* < 0.001 and *p* < 0.0001, respectively.

### 4.6. Field Experiments

Two field experiments were conducted under a contained facility at the Central Potato Research Institute (CPRI) Shimla, India. In the first field experiment, tuber sprouts from selective Désirée transgenic lines of each CC and the transgenic control (TC) were planted in the field on 9 June 2021, and grown for 12 weeks (i.e., until 10 September 2021). The second field experiment was conducted from 7–12 October 2022 (i.e., 19 weeks). The plants (transformed control and transgenic Désirée CC lines) were harvested post-senescence and the tuber parameters were recorded. For the first field experiment (2021), the average daily temperature and relative humidity were 22.5 °C and 73.5%, respectively. During the second field experiment (2022), the average daily temperature and relative humidity were 21.74 °C and 70.0%, respectively.

## 5. Conclusions

To the best of our knowledge, this is the first report that explores the use of multi-gene transformation tool for enhancing tuber productivity in potato. Using a multi-gene stacking approach, we demonstrate the cumulative effects of key mobile RNA (*StBEL5*) and its RNA-binding proteins (StPTB1 and StPTB6) driven by their respective native promoters in tandem, enhances tuber productivity. This approach could be adopted to other crops whose agronomic traits are governed by mobile macromolecules.

## Data Availability

All the data described in this study is included in the main figures.

## Supplementary Materials

The following supporting information can be downloaded at: https://www.mdpi.com/article/10.3390/ijms242115754/s1.
