# Supplementary material for: Tandem Expression of a Mobile RNA and Its RNA-Binding Protein(s) Enhances Tuber Productivity in Potato
Source: Int J Mol Sci. 2023 Oct 30;24(21):15754. doi: 10.3390/ijms242115754 (PMC10647900; doi:10.3390/ijms242115754)
Supplement: Supplementary file 1 [file ijms-24-15754-s001.zip › ijms-2610476-Supplementary Figures 1-2 .pdf]

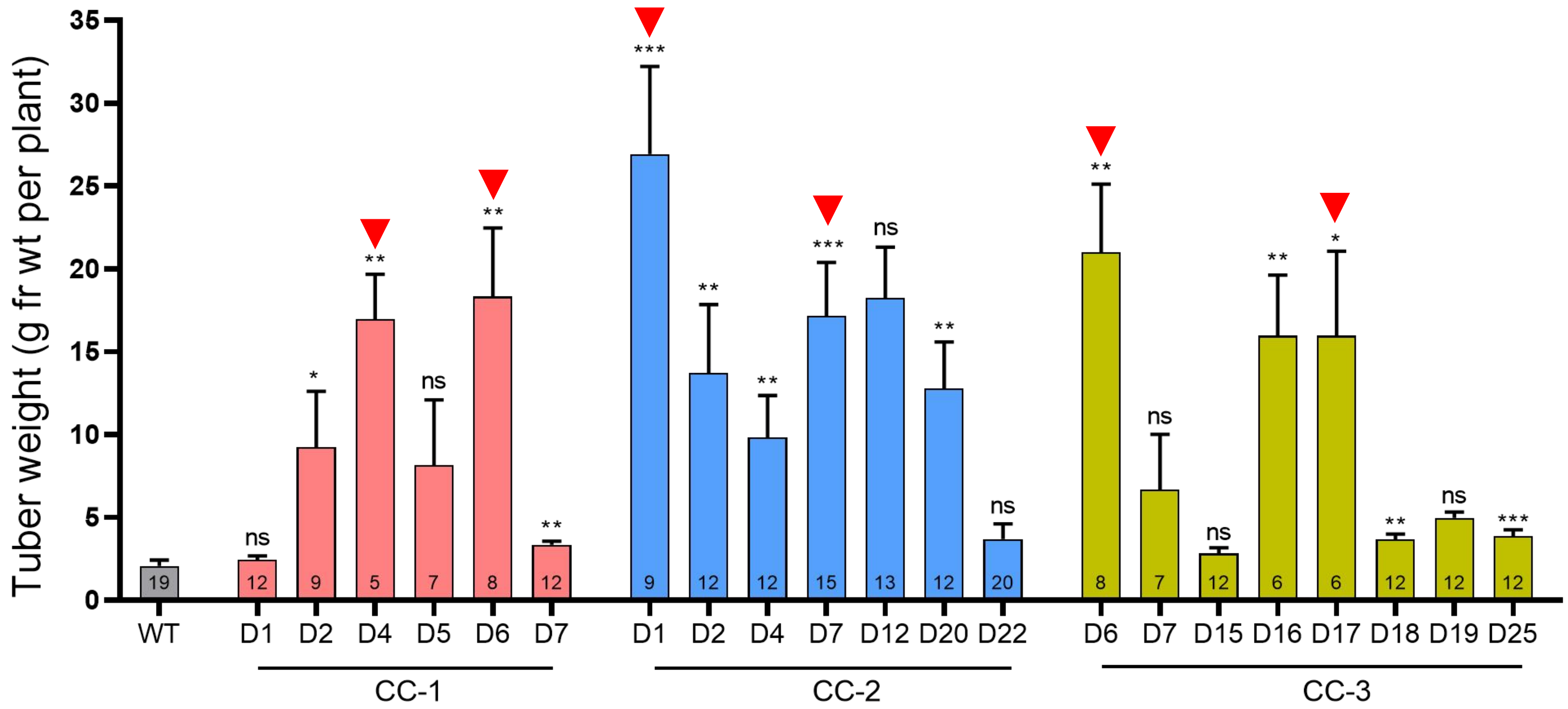

Figure S1. Average tuber weight in transgenic *Solanum tuberosum* cv. Désirée CC lines grown for two months in greenhouse under long-day conditions. Numbers inside the bars represent the number of plants per line (n). Error bars represent mean ±SEM. Student's t-test was used at p<0.05 (*P < 0.05, **P < 0.01, ***P < 0.001, ns, not significant). CC lines marked with red arrowhead were selected for further studies. We refer CC-1 lines (D4 and D6), CC-2 lines (D1 and D7), and CC-3 lines (D6 and D17) as respective #1 and #2 lines.

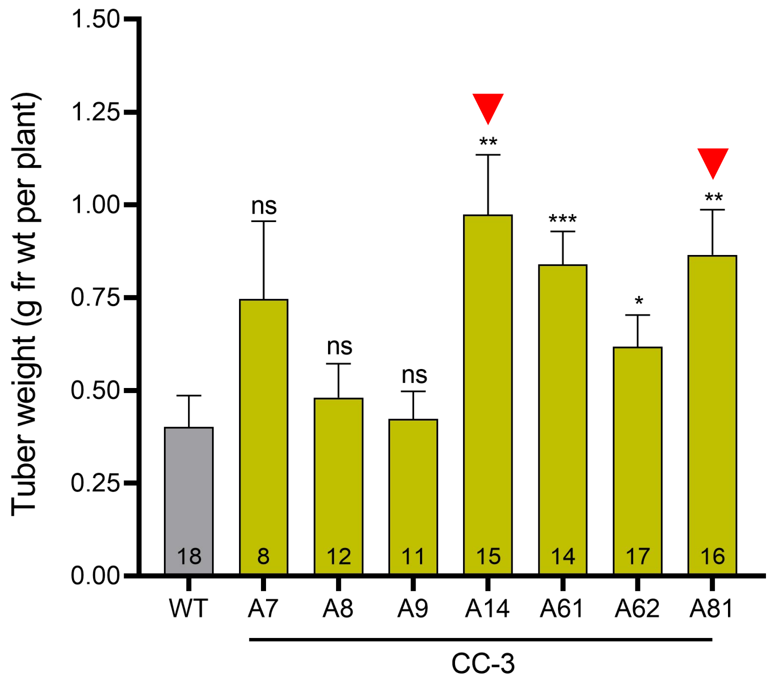

Figure S2. Average tuber weight in transgenic *Solanum tuberosum* ssp. *andigena.* CC lines grown in plant growth chamber. The plants were first grown for 10-weeks under long-day conditions, following which they were subjected to a short-day photoperiod induction for three weeks before harvesting the plants for tuber data. Numbers inside the bars represent the number of plants per line (n). Error bars represent mean ±SEM. Student's t-test was used at $p<0.05$ (*P < 0.05, **P < 0.01, ***P < 0.001， ns, not significant). In the main figure and the manuscript text hereafter, CC-3 lines marked with red arrowhead (A14 and A81) are referred as #1 and #2 line, respectively.
