# Supplementary material for: Tandem Expression of a Mobile RNA and Its RNA-Binding Protein(s) Enhances Tuber Productivity in Potato
Source: Int J Mol Sci. 2023 Oct 30;24(21):15754. doi: 10.3390/ijms242115754 (PMC10647900; doi:10.3390/ijms242115754)
Supplement: Supplementary file 1 [file ijms-24-15754-s001.zip › ijms-2610476-Supplementary tables.pdf]

Supplementary Table S1. List of primers used in this study.

Sr. No.	Primer name	Sequence (5'→3')	Amplicon size	Purpose
1	Prom*StBEL5* F	GCACCATAGCCAAAAAGTCTTAAGAC	2242 bp	*StBEL5* promoter cloning
2	Prom*StBEL5* R	CTGTAAACAGAAAGTAACAAAGG		
3	*StBEL5* CDS-5'UTR F	CATGCAGAGATAAAAATATAGATC	2716 bp	*StBEL5* coding sequence cloning
4	*StBEL5* CDS R	GCTAATCTAATAATGATAGCACA		
5	Prom*StPTB1* F	CTTGGGTTAGTCTTCCGCTTGTAG	2301 bp	*StPTB1* promoter cloning
6	Prom*StPTB1*-5'UTR R	TTATACCTGAATCACAACAAAAC		
7	*StPTB1* CDS F	GGTATAAATGTCTGATCCTTC	1338 bp	*StPTB1* coding sequence cloning
8	*StPTB1* CDS R	GACTTCTCAGATGCTCTGTAAC		
9	Prom*StPTB6* F	CGAGTTGTGGTGAGACGGTTAGTAG	2458 bp	*StPTB6* promoter cloning
10	Prom*StPTB6*-5'UTR R	TTTTTACCTGAATCACAAAAACAATG		
11	*StPTB6* CDS F	GGTAAAAAATGACTGAGCCGTC	1348 bp	*StPTB6* coding sequence cloning
12	*StPTB6* CDS R	GGCTTCTCAGATGCTTTGCAC		
13	*StBEL5*_3' F	TGTGATATTGTTCCTCTCAATTTGC	190 bp with *UbiTscr* R	Confirmation of stable transgenic lines
14	*StPTB1*_3' F	GAGGCACTCGTATGCAAAAA	157 bp with *NosTscr* R	
15	*StPTB6*_3' F	CATGCTACATCCCTTGGTGG	181 bp with *UbiTscr* R	
16	*NosTscr* R	GCAACAGGATTCAATCTTAAG		
17	*UbiTscr* R	ATTACCGTTCGACAAAAAGAGAAA		
18	*StEIF3e* qF	GGAGCACAGGAGAAGATGAAGGAG	164 bp	RT-qPCR analysis
19	*StEIF3e* qR	CGTTGGTGAATGCGGCAGTAGG		
20	*StBEL5* qF	AGGGATACTCTTATATTGTGTGTGAG	90 bp	
21	*StBEL5* qR	GTCTCCACTTCTTCTCTCCTATG		
22	*StCDF1* qF	TGCAGACTCGTCGATTGAAC	130 bp	

23	*StCDF1* qR	GAGTGCCTTTTCCTCACTCG		
24	*StPTB1* qF	GCGCAATAAACCAGATCATGC	112 bp	
25	*StPTB1* qR	GTTGACTTCCAGACGCTTTTC		
26	*StPTB6* qF	TCATGCTCTTGTTCAGATGGG	130 bp	
27	*StPTB6* qR	TGGTCCGGTGTTAATATTTGGG		
28	*StSWEET11b* qF	GTGATGCATGTGCATGTTTG	114 bp	
29	*StSWEET11b* qR	CAACGGCCAATCTCCTCTAA		
30	*StIT* qF	AAGAGCTCGTCGCCGTTAAA	150 bp	
31	*StIT* qR	GCAGGTCTACGAGCTGTGTT		
32	*StSP6A* qF	GACGATCTTCGCAACTTTTACA	74 bp	
33	*StSP6A* qR	CCTCAAGTTAGGGTCGCTTG		
34	*StSP5G* qF	GGTGTGTAGACTTTGGTGTGGTTT	96 bp	
35	*StSP5G* qR	GGCCTCAAGGCACATCCAT		
36	*StSP3D* qF	GGACCCAGATGCTCCAAGTC	64 bp	
37	*StSP3D* qR	CTTGCCAAAACTTGAACCTG		
38	*StPIN4* qF	GGGACCCACTGGACTGACTA	109 bp	
39	*StPIN4* qR	ACTTGCTGGAGGCATCTGTT		
40	*StGA2ox1* qF	TTCTCTACAATGAGTTCACATGGTC	78 bp	
41	*StGA2ox1* qR	GGGACAACCTATTATCACCAAGC		
42	*StCO2* qF	CTGGGTTTAGATAGAGAAGCAAG	177 bp	
43	*StCO2* qR	GTCAACGTCATCAAACTCGC		
44	*St14-3-3* qF	ACATTCCCAGAGTTCCTTGTCA	124 bp	
45	*St14-3-3* qR	ACTTTGGACCCAACCTGCTC		
